# Taxonomic classification of mental disorders

**DOI:** 10.1192/j.eurpsy.2022.1138

**Published:** 2022-09-01

**Authors:** M. Šablevičius

**Affiliations:** Private doctor‘s psychiatrist‘s practice, Private Doctor‘s Psychiatrist‘s Practice, Vilnius, Lithuania

**Keywords:** ICD-11, classification, DSM-5, ICD-10

## Abstract

**Introduction:**

DSM-5, ICD-10, and ICD-11 classifications can be described as „incoherent“. Psychopathology depends on “time of damage and resilience” ratio. Continuums of mental disorders compose a table, like a periodic table of chemical elements. Similar psychopathology can have different neurobiological origin, and vice versa.

**Objectives:**

Current classifications of mental disorders ICD-10, DSM-5, as well as the new ICD-11 being developed, do not show interrelations in pathogenesis between groups of mental disorders. This is a weak point of these classifications, although they serve a good purpose in relation to medical statistics and encoding requirements.

**Methods:**

Taxonomic classification of mental disorders proposed in this empirical study reveals interrelations between diagnostic categories of mental disorders. Classification as an object of this empirical study is initially developed on author’s observation of psychopathology in clinical practice. It also relies on scientific data of genetics and neurobiology of mental disorders.

**Results:**

The classification is based on two axes system. First axis reflects the time of damage of neural tissue in specific stage, i.e. neuron body genesis, neuron growths genesis, synaptic pruning or further neural information modeling. The second axis is connected with resilience. The two axes system includes in one continuum and connects into one classification table (** Figure 1**) almost all diagnostic groups from ICD-10 or DSM-5 (with two exclusions: “organic” type mental disorders and pathology of myelination process).

**Figure:**
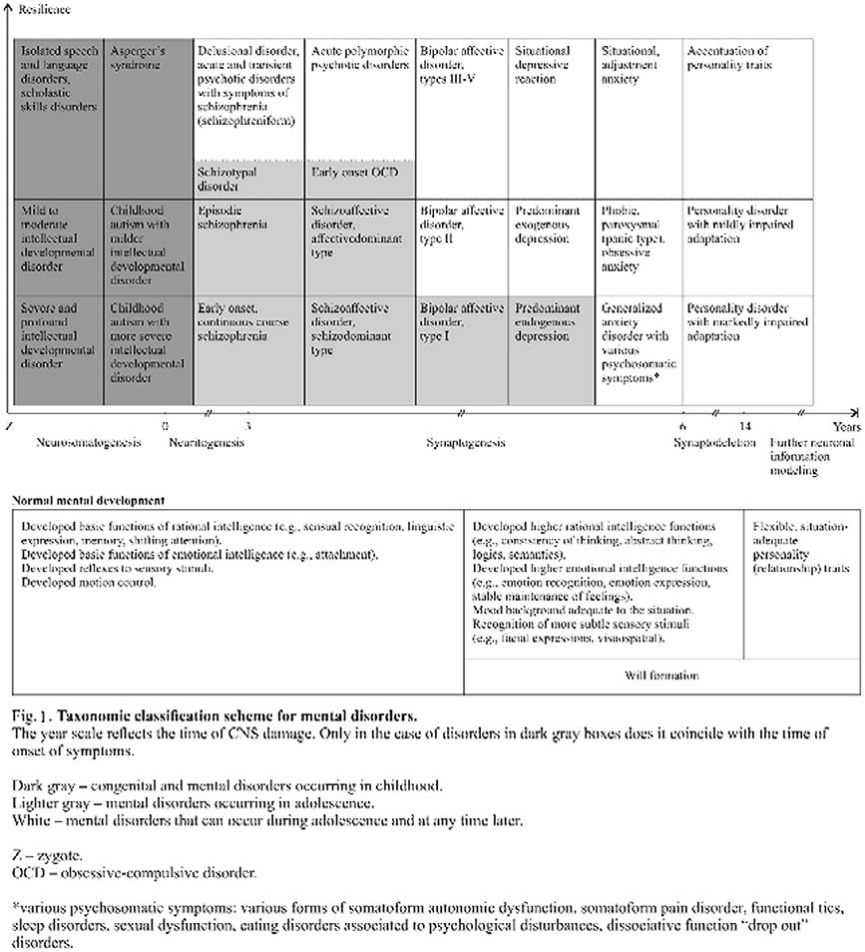

**Conclusions:**

This empirically derived concept of classification could be used in clinical practice in differential diagnosis, discovering heterogeneities in patients with same diagnostic “code”, planning treatment strategies, predicting course of mental disorders.

**Disclosure:**

No significant relationships.

